# Comparison of the 2018 and 2003 International Society of Nephrology/Renal Pathology Society classification in terms of renal prognosis in patients of lupus nephritis: a retrospective cohort study

**DOI:** 10.1186/s13075-020-02358-x

**Published:** 2020-11-04

**Authors:** Ryosuke Umeda, Soshiro Ogata, Shigeo Hara, Kazuo Takahashi, Daijo Inaguma, Midori Hasegawa, Hidetaka Yasuoka, Yukio Yuzawa, Hiroki Hayashi, Naotake Tsuboi

**Affiliations:** 1 Department of Nephrology, Fujita Health University School of Medicine, 1-98, Kutsukakecho Dengakugakubo, Toyoake City, Aichi 470-1192 Japan; 2 Department of Preventive Medicine and Epidemiology, National Cerebral and Cardiovascular Center, 6-1, Kisibesincho, Suita City, Osaka 564-8565 Japan; 3 Department of Pathology, Kobe City Medical Center General Hospital, 2-1-1, Minatojimaminamicho, Cyuo-ku, Kobe City, Hyogo 650-0047 Japan; 4 Department of Biomedical Molecular Sciences, Fujita Health University School of Medicine, Toyoake City, Japan; 5 Department of Rheumatology, Fujita Health University School of Medicine, 1-98, Kutsukakecho Dengakugakubo, Toyoake City, Aichi 470-1192 Japan

**Keywords:** Lupus nephritis, Systemic lupus erythematosus, The 2003 ISN/RPS classification, The 2018 revised ISN/RPS classification

## Abstract

**Background:**

Although the 2018 revised International Society of Nephrology/Renal Pathology Society (ISN/RPS) classification was proposed recently, until now, no reports have been made comparing the association of renal prognosis between the 2018 revised ISN/RPS classification and the 2003 ISN/RPS classification. The present study aimed to assess the usefulness, especially of activity and chronicity assessment, of the 2018 revised ISN/RPS classification for lupus nephritis (LN) in terms of renal prognosis compared to the classification in 2003.

**Methods:**

We retrospectively collected medical records of 170 LN patients from the database of renal biopsy at Fujita Health University from January 2003 to April 2019. Each renal biopsy specimen was reevaluated according to both the 2003 ISN/RPS classification and the 2018 revised ISN/RPS classification. Renal endpoint was defined as a 30% decline of estimated glomerular filtration rate (eGFR).

**Results:**

A total of 129 patients were class III/IV±V (class III, 44 patients; class IV, 35 patients; class III/IV+V, 50 patients). The mean age was 42 years, 88% were female, and the median observation period was 50.5 months. Renal prognosis was significantly different among the classes and significantly poor in the patients with higher modified National Institute of Health (mNIH) chronicity index (C index, ≥ 4) by a log-rank test (*p* = 0.05 and *p* = 0.02, respectively). By Cox proportional hazard models, only the C index was significantly associated with renal outcome (hazard ratio 1.32, 95% CI 1.11–1.56, *p* ≤ 0.01), while the classes, the 2003 activity and chronicity subdivision, and the mNIH activity index had no significant association with renal outcome. Each component of the C index was significantly associated with renal outcome in different models.

**Conclusion:**

This study demonstrates that the 2018 revised ISN/RPS classification was more useful in terms of association with renal prognosis compared to the 2003 ISN/RPS classification.

## Background

Systemic lupus erythematosus (SLE) is an autoimmune disease that affects many organ systems. Renal involvement is quite common (60–70%) among SLE patients, and it is associated with poor prognosis of the disease and higher mortality [1]. Early diagnosis and treatment of lupus nephritis (LN) are essential to preserving kidney function, and renal biopsy is recommended for the correct evaluation of renal pathological findings.

In 2003, in order to make some definitions of pathological findings clear and produce replicable results, the International Society of Nephrology/Renal Pathology Society (ISN/RPS) classification was proposed, which has since been widely accepted as the classification of LN [2, 3]. It was adopted by the following guidelines for the management of LN such as the American College of Rheumatology (ACR) [2], the Joint European League Against Rheumatism and the European Renal Association (EULAR/ERA-EDTA) [3, 4], the Kidney Disease: Improving Global Outcomes (KDIGO) working group [5], and the Japan College of Rheumatology [6].

However, this classification has several problems. For instance, definitions for some lesions such as endocapillary proliferation or fibrinoid necrosis are unclear. Furthermore, a meta-analysis study shows that there was no difference in renal prognosis between the S subclass and G subclass of class IV introduced in this revision [1, 7]. The classification is based only on glomerular lesion, and tubulointerstitial or vascular lesions are merely noted; on the other hand, tubulointerstitial lesions such as interstitial fibrosis and tubular atrophy are reported to be better predictors of estimated glomerular filtration rate (eGFR) decline than some active glomerular lesions [8]. Additionally, the designation of activity and chronicity through A, A/C, and C can be too broad of categorization, as details are not reflected.

In 2018, an international working group of leading nephropathologists proposed updates to the ISN/RPS classification system to solve such conflictions found in the classification proposed in 2003. In this new proposal, the definition of pathological findings was much more detailed and precise. Moreover, the subclass of class IV was removed. They also changed the method of evaluation of active and chronic status from shorthand A, A/C, and C subdivision to semiquantitative activity/chronicity index based on the National Institute of Health (NIH) index, labeled as modified NIH index (mNIH index) [9, 10].

There are several reports on the relationship between the prognosis of LN and the 2003 ISN/RPS classification or the original NIH index. For example, class IV LN [11], mixed proliferative and membranous LN [12], and high original NIH chronicity index (C index) score [13] are related to poor prognosis. However, until now, there have been few reports that investigate the prognostic value of the 2018 revised ISN/RPS classification or the mNIH index. Furthermore, no reports have been made comparing the association of renal prognosis between the 2018 revised ISN/RPS classification and the 2003 ISN/RPS classification. The present study aimed to confirm whether the classes, especially activity and chronicity subdivision in the 2003 ISN/RPS classification and NIH index in the 2018 revised ISN/RPS classification, are associated to renal prognosis among the histologically proven LN patients.

## Materials and methods

### Patients

**Fig. 1:**
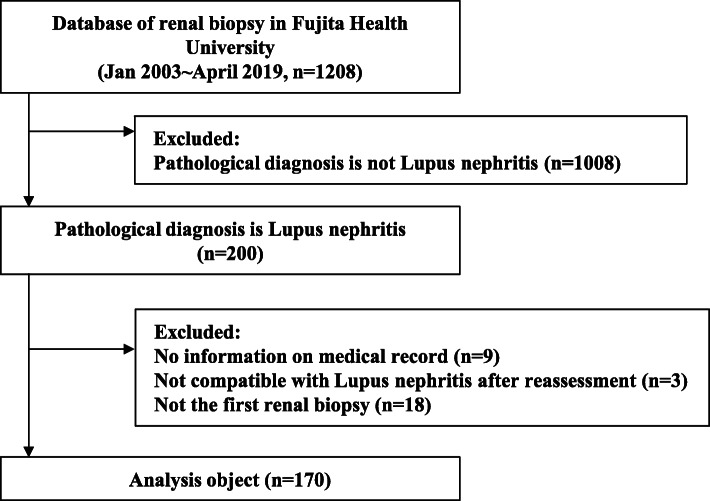
Patients’ flow chart

We used a retrospective cohort design and collected medical records of two hundred adult SLE patients from 1208 patients who underwent kidney biopsy at Fujita Health University from January 2003 to April 2019. From these, 9 cases not documented in the medical records and 3 cases of pathological findings unassociated with LN were excluded. For the 18 cases of patients who repeatedly received renal biopsies, data from the first renal biopsy of each case were used. Thus, a total of 170 cases were included for analysis (Fig. 1). All cases fulfilled ≥ 4 of the SLE classification criteria of the ACR in 1997 [14]. This study was conducted in accordance with the Declaration of Helsinki and approved by the Ethics Review Board of Fujita Health University (No. HM20-245) and opt-outed on its website.

### Histopathological studies

Renal biopsy specimens underwent light microscopy, immunofluorescence study, and electron microscopy study. Renal tissues were fixed in a 10% formalin neutral buffer solution, paraffin embedded, and sectioned at 1–3 μm thickness. Periodic acid–Schiff stain, periodic acid–methenamine silver stain, hematoxylin–eosin stain, and Masson’s trichrome stain were routinely performed. For immunofluorescent examination, parts of the tissues were embedded in optimal cutting temperature compound and quick-frozen in a dry ice–acetone mixture. After, they were sectioned at 3 μm by Tissue Tek POLAR-D (Sakura Seiki Co., Ltd., Japan) and stained with fluorescein isothiocyanate (FITC)-conjugated antihuman immunoglobulin (Ig) G, IgA, IgM, C3, C1q goat polyclonal antibodies (F. Hoffmann-La Roche Ltd., Switzerland), and FITC-conjugated antihuman C4 rabbit antibodies (DAKO., USA). As the evaluation was performed using an electron microscope, parts of the tissue were fixed in 2.5% glutaraldehyde and osmium tetroxide, embedded in Epon812 (OkenShoji Co., Tokyo, Japan), sectioned at 100 nm, stained with uranyl acetate and lead citrate, and observed with the electron microscope (JEM-1400Flash; JEOL Ltd., Tokyo, Japan).

All specimens contained more than 10 glomeruli and were each reevaluated using the 2003 ISN/RPS classification and the 2018 revised ISN/RPS classification [9, 15, 16]. For the activity and chronicity assessment, A, A/C, and C subdivisions were added to class III/IV±V in the 2003 ISN/RPS classification, and the mNIH index was added to all classes in the 2018 revised ISN/RPS classification. A summary of the components of the mNIH index is as follows: endocapillary hypercellularity, neutrophil infiltration or karyorrhexis, fibrinoid necrosis, hyaline deposits, cellular/fibrocellular crescent, and interstitial infiltration as active lesions, and glomerulosclerosis, fibrous crescent, tubular atrophy, and interstitial fibrosis as chronic lesions. Scores were assigned accordingly to the following percentages (0%, 0 points; < 25%, 1 point; 25~50%, 2 points; > 50%, 3 points): for endocapillary hypercellularity, neutrophil infiltration or karyorrhexis, fibrinoid necrosis, hyaline deposits, cellular/fibrocellular crescent, glomerulosclerosis, and fibrous crescent, the percentage of glomeruli with lesions out of the total number of glomeruli; for interstitial fibrosis and interstitial inflammation, the percentage of the cortex with lesions; and for tubular atrophy, the percentage of the cortical tubules with lesions. Scores were doubled for fibrinoid necrosis and cellular/fibrocellular crescent, and each of their total scores became the activity index (A index) and the C index. This contrasts with the original NIH activity and chronicity index in that the category including karyorrhexis is different and fibrocellular crescent is newly defined in the mNIH index. Pathological tissues were interpreted by experienced pathologist SH and experienced nephrologists RU and HH with medical records blinded. Readings by the pathologist and nephrologists were compared; if the result differed, a final decision was made upon consultation by the three.

### Clinical evaluation

Clinical data at the date of renal biopsy (age, sex, body mass index, baseline eGFR, serum creatinine [sCr], serum albumin, anti-phospholipid antibody, urinary protein to creatinine ratio [UPCR, g/g], duration between SLE diagnosis and renal biopsy) and longitudinal clinical examination data (sCr, eGFR, UPCR [g/g], medication) from the date of renal biopsy to April 2019 were extracted. eGFR was calculated using the equation for the Japanese population [17].

### Outcome

The primary outcome was set as the period from the date of renal biopsy to when GFR declined by 30%. When a patient reached the outcome or underwent renal replacement therapy or died or transferred to another hospital, data collection was terminated at that point. Patients usually took blood tests once every a few months, and eGFR was calculated.

### Statistical analysis

Baseline characteristics were summarized by mean (standard deviation [SD]) for continuous variables and *N* (%) for categorical variables. We plotted survival curves for the associations of the pathological features (i.e., classes, activity and chronicity subdivisions in 2003, and mNIH activity or chronicity index in 2018) with an eGFR decline of 30% using the Kaplan–Meier curve method and log-rank tests. The cutoff value of A index and C index was set to be its number of 80th percentile. We compared the pathological findings between mycophenolate mofetil (MMF)/cyclophosphamide (CYC)-treated patients and non-treated patients for those proportions by chi-squared tests and for those variables with ordinal scale by Mann–Whitney *U* tests. Additionally, the survival time of renal function decline was compared between those groups by the log-rank test. The reproducibility of class and each component of the mNIH index was analyzed by using intraclass correlation coefficient, kappa values, or weighted kappa values.

To investigate associations of pathological features with the primary outcome, we used Cox proportional hazard models. In the models, eGFR decline by 30% was modeled as a dependent variable, and pathological features were modeled as independent variables (i.e., classes, activity and chronicity subdivision in 2003, mNIH activity or chronicity index in 2018). We obtained hazard ratios (HR) and 95% confidence intervals (CI) that were crude in one model (i.e., non-adjusted; model 1) and adjusted for potential confounders in two models (i.e., model 2 and model 3). Model 2 was adjusted for age, sex, eGFR, and UPCR. Model 3 was adjusted for age, sex, eGFR, UPCR, duration of systemic lupus erythematosus, use of CYC or MMF, use of renin–angiotensin–aldosterone inhibitor, presence of nephrotic syndrome (NS), and presence of rapidly progressive glomerulonephritis (RPGN). Additionally, we investigated associations of each component of the chronicity index with the 30% eGFR decline using Cox proportional hazard models in which each component of the chronicity index was separately analyzed (i.e., these were analyzed in different models).

The significance tests were two-sided, and the significance level for all analyses was *p* value < 0.05. When there were missing values of a potential confounder, they were excluded from the analyses. We used the R statistical software (R Core Team (2020). R: a language and environment for statistical computing. R Foundation for Statistical Computing, Vienna, Austria. URL https://www.R-project.org/.).

## Results

### Clinical characteristics of patients of LN

**Fig. 2:**
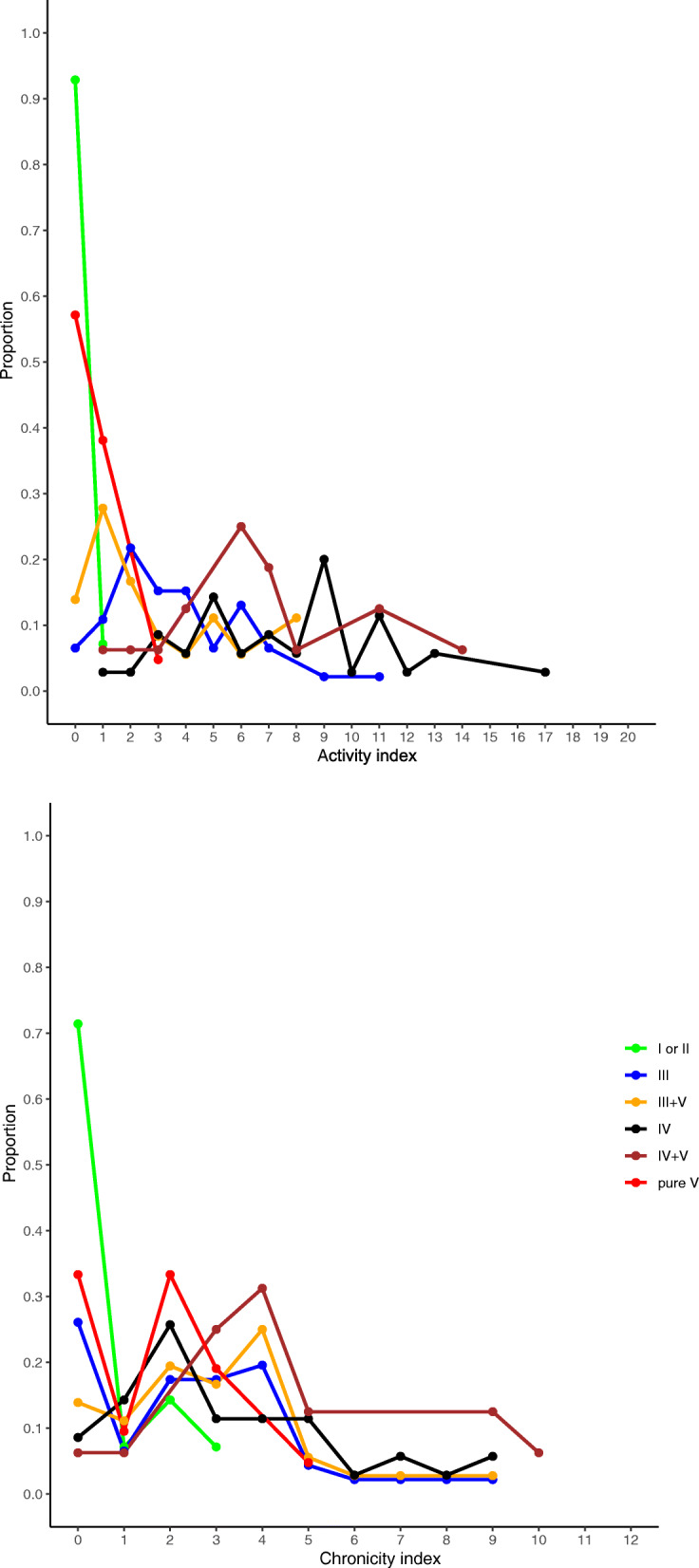
Frequency distribution chart of the modified National Institute of Health activity and chronicity index

The baseline data for proliferative LN (class III/IV±V) patients are shown in Table 1 (class III, 44 patients; class IV, 35 patients; and class III/IV+V, 50 patients). The age of patients ranged between 17 and 78 with a mean (SD) of 42 (14), and 88% were female. Baseline eGFR (mL/min/1.73 m^2^) and UPCR (g/g) were 79.5 (24.3) and 1.2 (1.3) in class III, 66.7 (27.0) and 3.2 (2.4) in class IV, and 82.9 (32.6) and 3.0 (3.1) in class III/IV+V, respectively. The percentage of patients with NS/RPGN was 18.2/9.1 in class III, 65.7/25.7 in class IV, and 50.0/6.0 in class III/IV+V. All the patients from the 2003 ISN/RPS classification matched with the 2018 revised ISN/RPS classification. The proportion of the subdivision of 2003 ISN/RPS classification was as follows: A was 25.6%, A/C was 54.3%, and C was 20.1%. The median (interquartile range (IQR)) for A index and C index of the mNIH index from the 2018 revised ISN/RPS classification was 3 (2, 5)/2 (2, 4) in class III, 8 (1, 6)/3 (2, 5) in class IV, and 3 (1, 6)/3 (2, 4) in class III/IV+V. The frequency distribution of the A index and C index is shown in Fig. 2, and all the baseline data including patients of classes I, II, and V are presented in Table 2.

**Table 1:** Baseline characteristics of patients of proliferative lupus nephritis

	III	IV	III/IV+V
Number	44	35	50
Age, years, mean ± SD	45.7 (15.3)	40.0 (14.1)	41.1 (11.6)
Sex, female, *n* (%)	41 (93.2)	32 (91.4)	40 (80.0)
BMI, mean ± SD	22.0 (3.5)	21.8 (4.0)	21.5 (3.8)
Duration of SLE, years, mean ± SD	10.9 (9.7)	10.5 (7.9)	12.1 (6.6)
Systolic BP, mmHg, mean ± SD	128.0 (18.0)	137.7 (25.7)	128.3 (24.0)
Serum creatinine, mg/dL, mean ± SD	0.8 (0.3)	0.9 (0.5)	1.0 (0.6)
Antiphospholipid antibody, *n* (%)	18 (40.9)	12 (34.3)	12 (24.0)
eGFR, mL/min/1.73 m^2^, mean ± SD	79.5 (24.3)	66.7 (27.0)	82.9 (32.6)
Urinary protein, g/g, mean ± SD	1.2 (1.3)	3.2 (2.4)	3.0 (3.1)
Nephrotic syndrome, *n* (%)	8 (18.2)	23 (65.7)	25 (50.0)
RPGN, *n* (%)	4 (9.1)	9 (25.7)	3 (6.0)
Activity and chronicity assessment			
ISN/RPS 2003 classification, *n* (%)			
A	18 (40.9)	8 (22.9)	7 (14.0)
A/C	20 (45.5)	22 (62.9)	28 (56.0)
C	6 (13.6)	5 (14.3)	15 (30.0)
2018 mNIH index, median (IQR)			
Activity index	3 (2, 5)	8 (1, 6)	3 (1, 6)
Chronicity index	2 (2, 4)	3 (2, 5)	3 (2, 4)
Treatment			
Initial dosage of PSL, mean ± SD	39.3 (15.5)	42.8 (15.5)	38.3 (15.5)
CYC or MMF, *n* (%)	8 (18.2)	16 (45.7)	8 (16.0)
Other immunosuppressants, *n* (%)	11 (25.0)	15 (42.9)	14 (28.0)
RAS inhibitor, *n* (%)	14 (31.8)	15 (42.9)	16 (32.0)

*SD* standard deviation, *BMI* body mass index, *BP* blood pressure, *SLE* systemic lupus erythematosus, *EGFR* estimated glomerular filtration rate, *RPGN* rapidly progressive glomerulonephritis, *ISN/RPS* International Society of Nephrology/Renal Pathology Society, *IQR* interquartile range, *mNIH* modified National Institute of Health, *PSL* prednisolone, *CYC* cyclophosphamide, *MMF* mycophenolate mofetil, *RAS* renin–angiotensin–aldosterone system

**Table 2:** Baseline characteristics of patients of all classes of lupus nephritis

	I/II	III	III+V	IV	IV+V	pure V
Number	14	46	36	35	16	21
Age, years, mean ± SD	35.4 (15.2)	44.9 (15.4)	41.8 (12.8)	40.0 (14.1)	40.7 (10.6)	39.7 (13.9)
Sex, female, *n* (%)	9 (64.3)	43 (93.5)	28 (77.8)	32 (91.4)	13 (81.2)	15 (71.4)
BMI, mean ± SD	20.8 (2.8)	21.9 (3.5)	21.5 (3.8)	21.8 (4.0)	21.9 (3.8)	21.3 (3.1)
Duration of SLE, years, mean ± SD	9.4 (8.6)	10.6 (9.6)	11.9 (6.3)	10.5 (7.9)	12.7 (6.7)	8.3 (3.8)
Systolic BP, mmHg, mean ± SD	122.6 (16.7)	126.8 (18.4)	122.0 (20.4)	137.7 (25.7)	130.0 (27.1)	118.7 (17.5)
Serum Cr, mg/dL, mean ± SD	0.66 (0.19)	0.80 (0.30)	0.87 (0.62)	0.90 (0.49)	1.17 (0.62)	0.73 (0.35)
Antiphospholipid antibody, *n* (%)	1 (7.1)	19 (41.3)	9 (25.0)	12 (34.3)	3 (18.8)	7 (33.3)
eGFR, mL/min/1.73 m^2^, mean ± SD	114.9 (35.8)	79.5 (24.3)	91.8 (30.3)	66.7 (27.0)	63.8 (29.7)	98.6 (35.7)
Urinary protein, g/g, mean ± SD	0.83 (0.89)	1.19 (1.28)	2.80 (2.58)	3.22 (2.36)	3.77 (4.03)	1.48 (1.76)
Nephrotic syndrome, *n* (%)	1 (7.1)	8 (17.4)	17 (47.2)	23 (65.7)	9 (56.2)	4 (19.0)
RPGN, *n* (%)	0 (0.0)	5 (10.9)	1 (2.8)	9 (25.7)	2 (12.5)	0 (0.0)
Activity and chronicity assessment						
ISN/RPS 2003 classification, *n* (%)						
A	0 (0.0)	18 (39.1)	6 (16.7)	8 (22.9)	1 (6.2)	0 (0.0)
A/C	0 (0.0)	22 (47.8)	17 (47.2)	22 (62.9)	12 (75.0)	0 (0.0)
C	14 (100)	6 (13.0)	13 (36.1)	5 (14.3)	3 (18.8)	21 (100)
2018 mNIH index, median (IQR)						
Activity index	0 (0, 0)	3 (2, 5)	2 (1, 5)	8 (1, 6)	6 (4, 79)	0 (0, 1)
Chronicity index	0 (0, 0.75)	2 (2, 4)	3 (1.5, 4)	3 (2, 5)	4 (3, 5)	2 (0, 3)
Treatment, *n* (%)						
CYC or MMF	2 (15.4)	8 (17.4)	4 (11.1)	16 (45.7)	4 (25.0)	2 (9.5)
RAS inhibitor	3 (23.1)	14 (30.4)	11 (30.6)	15 (42.9)	7 (43.8)	4 (19.0)

*SD* standard deviation, *BMI* body mass index, *BP* blood pressure, *Cr* creatinine, *SLE* systemic lupus erythematosus, *EGFR* estimated glomerular filtration rate, *RPGN* rapidly progressive glomerulonephritis, *ISN/RPS* International Society of Nephrology/Renal Pathology Society, *IQR* interquartile range, *mNIH*, modified National Institute of Health, *CYC* cyclophosphamide, *MMF* mycophenolate mofetil, *RAS* renin–angiotensin–aldosterone system, *ref* reference

### Pathological findings and renal prognosis

**Fig. 3:**
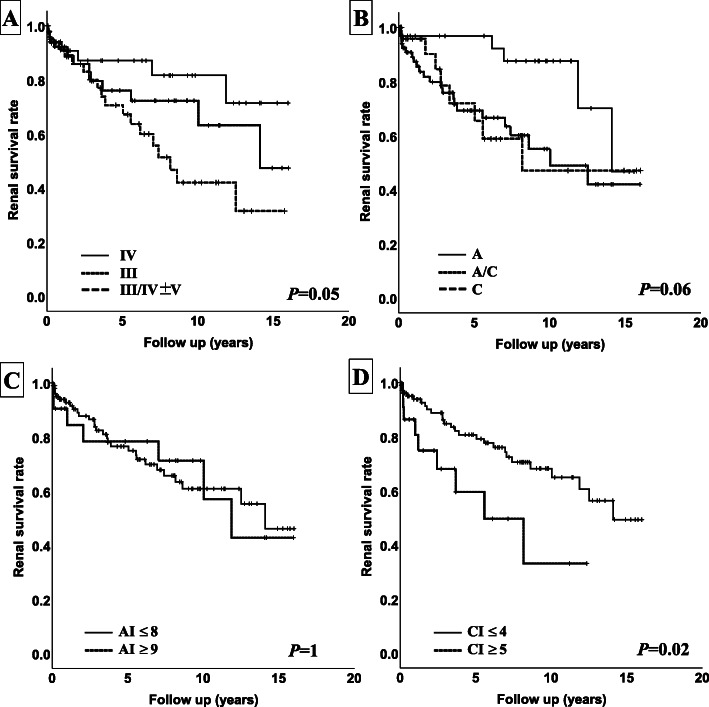
Kaplan–Meier analysis with a 30% decline of eGFR in patients of proliferative lupus nephritis. **a** Class (III, IV, III/IV+V). **b** 2003 activity and chronicity assessment (A, A/C, C). **c** Modified National Institute of Health Activity index (AI ≤ 8, 9 ≤ AI). **d** Modified National Institute of Health chronicity index (CI ≤ 4, 5 ≤ CI). AI, activity index; CI, chronicity index

Survival curves using the Kaplan–Meier curves method and log-rank tests, and a multivariate analysis using Cox proportional hazard models for the associations of eGFR decline by 30% and the pathological features (class, activity and chronicity subdivisions in the 2003 classification, and mNIH index) were shown in Fig. 3 and Table 3, respectively. Renal prognosis was significantly different among the classes by a log-rank test (Fig. 3a), while there was no significant difference by Cox proportional hazard models (Table 3). There was also no significant difference in renal outcome among the activity and chronicity subdivisions of A, A/C, and C by a log-rank test (Fig. 3b). Although subdivision A/C showed a significant association to renal outcomes in model 1 (HR, 3.08; 95% CI, 1.12–8.44; *p* = 0.03), the difference was not seen in models 2 and 3 (HR 2.67, 95% CI 0.84–8.49, *p* = 0.10 in model 2; Table 3). There was no difference among the two A index groups (≥ 8 or ≤ 9) in the log-rank test (Fig. 3c) and no association in outcome by Cox proportional hazard models (Table 3). On the other hand, there was a significant difference among the two groups of the C index (≤ 4 or ≥ 5) in the log-rank test (Fig. 3d), and the C index showed a significant association with renal prognosis by Cox proportional hazard models (HR 1.29, 95% CI 1.14–1.46, *p* ≤ 0.01 in model 1; HR 1.32, 95% CI 1.11–1.56, *p* ≤ 0.01 in model 2; HR 1.29, 95% CI 1.05–1.58, *p* = 0.01 in model 3).

**Table 3:** Associations between pathological features and eGFR decline by 30%; Cox proportional hazard models

	Model 1 (*n* = 129)			Model 2 (*n* = 128)			Model 3 (*n* = 128)		
	HR	95% CI	*p* value	HR	95% CI	*p* value	HR	95% CI	*p* value
2003 classification model									
Class									
III (ref)	1.00			1.00			1.00		
IV	0.46	(0.16–1.27)	0.13	0.76	(0.25–2.33)	0.63	0.83	(0.27–2.62)	0.76
III or IV+V	1.29	(0.60–2.79)	0.52	1.82	(0.67–4.89)	0.24	1.41	(0.49–4.02)	0.52
Activity/chronicity assessment									
A (ref)	1.00		1.00			1.00			
A/C	3.08	(1.12–8.44)	0.03	2.67	(0.84–8.49)	0.10	2.16	(0.64–7.34)	0.22
C	2.19	(0.68–7.00)	0.19	2.59	(0.71–9.47)	0.15	2.52	(0.66–9.61)	0.17
2018 classification model									
Class									
III (ref)	1.00			1.00			1.00		
IV	0.39	(0.12–1.21)	0.10	0.62	(0.19–2.00)	0.42	0.61	(0.18–2.09)	0.43
III or IV+V	1.35	(0.63–2.89)	0.44	1.68	(0.65–4.34)	0.28	1.30	(0.46–3.69)	0.62
2018 mNIH index									
Activity index	1.05	(0.93–1.17)	0.43	1.04	(0.92–1.18)	0.53	1.05	(0.92–1.19)	0.49
Chronicity index	1.29	(1.14–1.46)	< 0.01	1.32	(1.11–1.56)	< 0.01	1.29	(1.05–1.58)	0.01
Activity and chronicity index	1.02	(0.97–1.07)	0.52	1.01	(0.96–1.07)	0.72	1.02	(0.97–1.08)	0.44

Model 1, not adjusted; model 2, adjusted for age, sex, estimated glomerular filtration rate, and urinary protein; model 3, adjusted for age, sex, estimated glomerular filtration rate, urinary protein, duration of systemic lupus erythematosus, use of cyclophosphamide or mycophenolate mofetil, use of renin–angiotensin–aldosterone inhibitor, nephrotic syndrome or not, and rapidly progressive glomerulonephritis or not

*HR* hazard ratio, *CI* confidence interval, *mNIH* modified National Institute of Health, *ref* reference

**Fig. 4:**
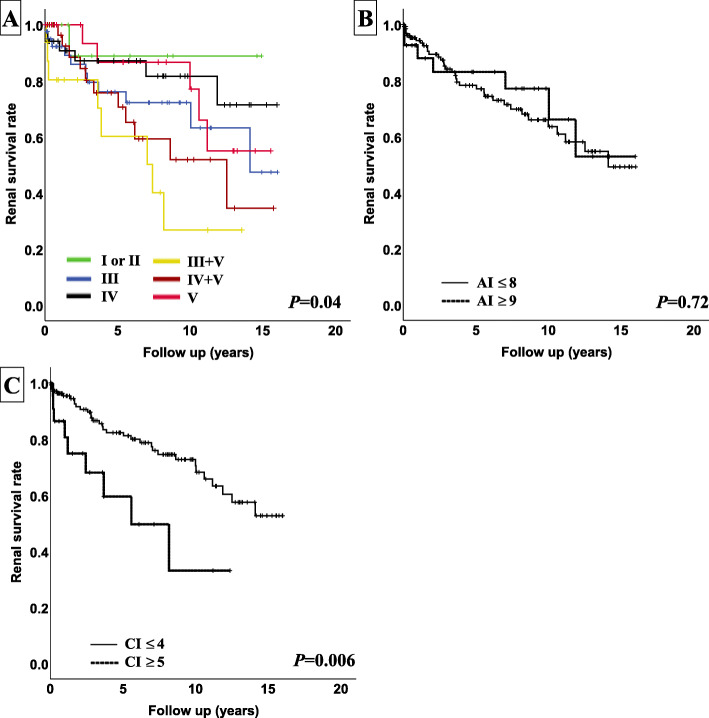
Kaplan–Meier analysis with a 30% decline of eGFR in patients of all classes. **a** Class (I or II, III, IV, III+V, IV+V, V). **b** Modified National Institute of Health Activity index (AI ≤ 8, 9 ≤ AI). **c** Modified National Institute of Health chronicity index (CI ≤ 4, 5 ≤ CI)

Survival curves using the Kaplan–Meier curves method and multivariate analysis using Cox proportional hazard models of patients of all classes including classes I, II, and V are shown in Fig. 4 and Table 4. Although a significant difference was not seen among the A index groups (≥  8 or ≤ 9), classes (I/II, III, IV, III+V, IV+V, V) and C index (≤ 4 or ≥ 5) were significantly associated with renal outcome in the log-rank test. By Cox proportional hazard models, only the C index had a significant association with renal outcome (HR 1.28, 95% CI 1.09–1.50, *p* ≤ 0.01).

**Table 4:** Associations between pathological features and eGFR decline by 30%; Cox proportional hazard models (*n* = 162)

	HR	95% CI	*p* value
Class			
I or II (ref)	1.00		
III	1.25	(0.12–12.86)	0.85
IV	0.72	(0.05–9.64)	0.80
III+V	2.07	(0.21–20.34)	0.53
IV+V	3.00	(0.23–38.81)	0.40
V	1.47	(0.16–13.30)	0.73
2018 mNIH index			
Activity index	1.04	(0.91–1.18)	0.55
Chronicity index	1.28	(1.09–1.50)	< 0.01
Activity/chronicity index	1.00	(0.95–1.05)	0.94

Those HRs were adjusted for age, sex, estimated glomerular filtration rate, and urinary protein

*HR* hazard ratio, *CI* confidence interval, *mNIH* modified National Institute of Health, *ref* reference

Cox proportional hazard models for the association of each component of the mNIH C index with renal outcome are shown in Table 5. Glomerular sclerosis, fibrous crescent, tubular atrophy, and interstitial fibrosis all showed significant associations with renal outcomes in model 2 (HR 1.94, 95% CI 1.11–3.39, *p* = 0.02; HR 2.04, 95% CI 1.03–4.03, *p* = 0.04; HR 1.68, 95% CI 1.04–2.71, *p* = 0.03; HR 2.01, 95% CI 1.27–3.20, *p* ≤ 0.01, respectively).

**Table 5:** Association of every component of chronicity index and eGFR decline by 30% (*n* = 128)

Component of chronicity index	HR	95% CI	*p* value
Glomerulosclerosis	1.94	(1.11–3.39)	0.02
Fibrous crescent	2.04	(1.03–4.03)	0.04
Tubular atrophy	1.68	(1.04–2.71)	0.03
Interstitial fibrosis	2.01	(1.27–3.20)	< 0.01

Hazard ratio was adjusted for age, sex, estimated glomerular filtration rate, urinary protein, class, and activity index. Each component of the chronicity index was analyzed in different models

*HR* hazard ratio, *CI* confidence interval

Then, we analyzed the reproducibility of class and each component of the mNIH index by using intraclass correlation coefficient, kappa values, or weighted kappa values (Table 6). All kappa values were higher than 0.8, showing good reproducibility. Note that this was not the reproducibility for pathological findings of each glomerulus, but the reproducibility of class and semiquantitative scoring of the mNIH index.

**Table 6:** ICC, kappa values, or weighted kappa values for the class and the components of the mNIH index

	Values
Class	0.840^a^
Activity index	0.962^b^
Endocapillary hypercellularity	0.889^c^
Neutrophils/karyorrhexis	0.958^c^
Fibrinoid necrosis	0.873^c^
Hyaline deposits	0.803^c^
Cellular/fibrocellular crescents	0.838^c^
Interstitial inflammation	0.878^c^
Chronicity index	0.955^b^
Glomerulosclerosis	0.835^c^
Fibrous crescents	0.927^c^
Tubular atrophy	0.902^c^
Interstitial fibrosis	0.806^c^

*mNIH* modified National Institute of Health, *ICC* intraclass correlation coefficient

^a^Kappa values

^b^Intraclass correlation coefficient

^c^Weighted kappa values

Since our cohort had a relativity low proportion of MMF/CYC usage, we analyzed the association between pathological findings and renal prognosis when divided into patients treated with or without MMF/CYC (Table 7). In the MMF/CYC group, the activity index was significantly high than the non-MMF/CYC-treated group (*p* = 0.02). There was a significant difference in class (*p* < 0.01), while there was no significant difference in renal outcome among the two groups.

**Table 7:** Relationship between pathological findings or renal outcome and use of MMF/CYC

	MMF/CYC (+)	MMF/CYC (−)	*p* value
Class, *n* (%)			< 0.01^a^
III	8 (18.2)	36 (82.0)	
IV	16 (45.7)	19 (54.3)	
III/IV+V	8 (16.0)	42 (84.0)	
Activity index, median (IQR)	6 (4, 9)	3 (2, 6)	0.02^b^
Chronicity index, median (IQR)	4 (2, 4)	3 (1, 4)	0.26^b^
eGFR 30% decline, *n* (%)	5 (15.6)	31 (30.7)	0.15^c^

*IQR* interquartile range, *MMF* mycophenolate mofetil, *CYC* cyclophosphamide

^a^Pearson’s chi-square test

^b^Mann–Whitney *U* test

^c^Log-rank test

## Discussion

In our study of this proliferative LN cohort, the mNIH C index from the 2018 revised classification showed a significant association of a 30% decrease of eGFR. The renal outcome was significantly associated with all C index components, including tubulointerstitial lesions not only glomerular lesions. On the other hand, there was no significant association between renal outcome and the activity and chronicity subdivisions based on the 2003 ISN/RPS classification. In terms of associations with renal prognosis, the activity and chronicity assessment based on the 2018 revised ISN/RPS classification was more useful compared to that of the 2003 ISN/RPS classification.

Tao et al. had already studied the association of the 2018 revised ISN/RPS classification with renal prognosis and reported that fibrous crescent, tubular atrophy/interstitial fibrosis, and the C index were associated with poor renal prognosis [18]. Our study also showed significant associations of the C index and its components including fibrous crescent, tubular atrophy, and interstitial fibrosis by a 30% decrease of eGFR, which supported the previous study. In this study, we further investigated the usefulness of the 2018 revised ISN/RPS classification in terms of the association with renal prognosis compared to the 2003 ISN/RPS classification by reclassifying the patients of LN using both classification criteria. Such reports had not been made until now, making this the first report of its kind.

In this study, the mNIH C index showed a significant association by a 30% decrease of eGFR, although it was not shown in the activity and chronicity subdivisions in 2003. The most important difference in activity and chronicity assessment between the 2003 and 2018 ISN/RPS classifications is the inclusion of the evaluation for tubulointerstitial lesions. This is because tubulointerstitial lesions, not only glomerular lesions, have shown to be significantly associated with renal prognosis of LN in previous studies [8, 13, 19, 20]. It is known that whatever the causes of chronic kidney disease (CKD) are CKD gradually exacerbates tubulointerstitial hypoxia by multifactorial mechanisms such as loss of peritubular capillaries, decreased oxygen diffusion by fibrosis, or decreasing of blood flow by glomerulosclerosis. As a result of tubulointerstitial hypoxia, CKD progresses, and it forms a malignant cycle. It is known as the “final common pathway” [21]. We should recognize tubulointerstitial lesions as important prognostic factors even in a glomerular disease. Actually, in IgA nephropathy, a common form of glomerulonephritis, interstitial fibrosis, and tubular atrophy is shown to be significant lesions that strongly associate with renal prognosis [22]. In this context, the adoption of evaluation of tubulointerstitial lesions in the classification of LN should improve the predictability of the renal outcome.

As explanations for why chronicity assessment of the 2003 ISN/RPS classification showed no significant association with renal prognosis, in addition to the absence of the evaluation for tubulointerstitial lesions mentioned above, there is a problem in the designation of subdivisions. Chronicity subdivisions in the 2003 classification are not quantitative. Patients with a single C lesion and patients with diffuse C lesions are classified into the same C subdivision. In A/C subdivision, whether the active lesion is dominant, or the chronic lesion is dominant, is not expressed. Subdivision of A/C occupies a large proportion (54.3% in our cohort), and they are formed as a heterogeneous group. Hiramatsu et al. have reported that class IV-G (A/C) patients with diffuse C lesions are more likely to reach the outcome of the doubling of sCr than class IV-G (A/C) patients with focal C lesions [23]. This study showed the importance of quantitative chronic lesions in the prediction of renal prognosis.

Meanwhile, the A index was not significantly associated with renal outcome in our study. Austin et al. showed that patients with high A index had a strong association with ESRD [10], but no significant associations have been shown in recent reports [11, 13, 19]. In recent years, the treatment regimen of glucocorticoid with other immunosuppressants including CYC or MMF for the remission induction would sufficiently improve some components of the A index [24]. The A index may be less important as a renal prognostic factor in this era.

The mNIH index of the 2018 revised classification is proposed to be applied to all classes, unlike the activity and chronicity subdivisions of the 2003 ISN/RPS classification which are limited to class III/IV±V patients. Although many patients in classes I, II, and V had a low A index and C index, some patients had a relatively high C index. In the multivariate analysis using Cox proportional hazard models in all classes, the C index was also shown to have significant associations with renal prognosis. Even in class II or pure class V, for cases with high C index, careful observation and follow-up on renal function may be desirable. Additionally, we set a 30% GFR decline as the primary endpoint. Although the arrival of doubling sCr, which is strongly associated with subsequent risk of ESRD, has been widely accepted as the renal outcome, it is a late event. In fact, in this study, a 30% GFR decline was observed in 36 patients, while the doubling of sCr and ESRD was observed only in 13 and 3 patients, respectively. It is shown that 30% eGFR decline, in replacement of sCr doubling, is effective as an early predictive marker of CKD progression [25].

There are several limitations. First, this study is a retrospective observational study. Second, the nationality and race are limited to mostly Japanese. Third, MMF or CYC as the current standard drug for proliferative LN was only used for 25% of patients in our cohort. This is because MMF or CYC was recently approved for the treatment of LN as health insurance treatment in Japan.

The strength of this study is that compared to SLE patient cohorts of previous reports, there are more patients in this study. In addition, this is the first report that investigates the usefulness of the 2018 revised ISN/RPS classification compared to the 2003 ISN/RPS classification in terms of the association with renal prognosis.

While the activity and chronicity subdivisions of the 2003 ISN/RPS classification showed no association with renal prognosis, the mNIH C index of the 2018 revised classification showed a significant association by a 30% eGFR decrease. Each category of the C index was independently associated with poor renal prognosis. In terms of associations with renal prognosis, the 2018 activity and chronicity assessment was more useful compared to the 2003 activity and chronicity assessment.

## Data Availability

The datasets supporting the conclusions of this article are included within the article.

## Abbreviations

LN: Lupus nephritis
ISN/RPS: International Society of Nephrology/Renal Pathology Society
eGFR: Estimated glomerular filtration rate
mNIH: Modified National Institute of Health
A index: Activity index
C index: Chronicity index
SLE: Systemic lupus erythematosus
ACR: The American College of Rheumatology
EULAR/ERA-EDTA: The Joint European League Against Rheumatism and the European Renal Association
KDIGO: Kidney Disease: Improving Global Outcomes
FITC: Fluorescein isothiocyanate
Ig: Immunoglobulin
sCr: Serum creatinine
UPCR: Urinary protein to creatinine ratio
SD: Standard deviation
CI: Confidence intervals
CYC: Cyclophosphamide
MMF: Mycophenolate mofetil
NS: Nephrotic syndrome
RPGN: Rapidly progressive glomerulonephritis
IQR: Interquartile range
CKD: Chronic kidney disease
ICC: Intraclass correlation coefficient
